# Chromosomal changes during development and progression of prostate adenocarcinomas

**DOI:** 10.1054/bjoc.2000.1533

**Published:** 2001-01

**Authors:** H Zitzelsberger, D Engert, A Walch, U Kulka, M Aubele, H Höfler, M Bauchinger, M Werner

**Affiliations:** Institute of Radiobiology, GSF-Forschungszentrum für Umwelt und Gesundheit GmbH, Ingolstäder Landstr. 1, Neuherberg, D-85764, Germany; Institute of Pathology, Technische Universität, Ismaninger Str. 22, München, D-81675, Germany; Institute of Pathology, GSF-Forschungszentrum für Umwelt und Gesundheit GmbH, Ingolstädter Landstr. 1, Neuherberg, D-85764, Germany; Institute of Radiation Biology, Ludwig Maximilians Universität, Schillerstr. 42, München, D-80336, Germany

**Keywords:** prostate carcinoma, prostatic intraepithelial neoplasia, tumour progression, comparative genomic hybridization

## Abstract

Chromosomal copy number changes were investigated in 16 prostate carcinomas, 12 prostatic intraepithelial neoplasias (PIN; 4 low-grade and 8 high-grade) adjacent to the invasive tumour areas, and 5 regional lymph node metastases. For this purpose, comparative genomic hybridization (CGH) was performed and a copy number karyotype for each histomorphological entity was created. CGH on microdissected cells from non-neoplastic glands was carried out on 3 different cases to demonstrate the reliability of the overall procedure. None of the non-neoplastic tissue samples revealed chromosome copy number changes. In PIN areas, chromosomal imbalances were detected on chromosomes 7, 8q, Xq (gains), and on 4q, 5q, 8p, 13q and 18q (losses). In the primary tumours, recurrent (at least 25% of cases) gains on chromosomes 12q and 15q, and losses on 2q, 4q, 5q, Xq, 13q and 18q became apparent. Losses on 8p and 6q as well as gains on 8q and of chromosome 7 were also detected at lower frequencies than previously reported. The pooled CGH data from the primary carcinomas revealed a novel region of chromosomal loss on 4q which is also frequently affected in other tumour entities like oesophageal adenocarcinomas and is supposed to harbour a new tumour suppressor gene. Gains on chromosome 9q and of chromosome 16 and loss on chromosome 13q were observed as common aberrations in metastases and primary tumours. These CGH results indicate an accumulation of chromosomal imbalances during the PIN–carcinoma–metastasis sequence and an early origin of tumour-specific aberrations in PIN areas. © 2001 Cancer Research Campaign http://www.bjcancer.com


*British Journal of Cancer* (2001) **84**(2), 202–208
© 2001 Cancer Research Campaign
doi: 10.1054/ bjoc.2000.1533, available online at http://www.idealibrary.com on IDEAL®

http://www.bjcancer.com

### Chromosomal changes during development and progression of prostate adenocarcinomas

**H Zitzelsberger[1,4], D Engert[1], A Walch[2], U Kulka[1], M Aubele[2], H Höfler[2,3], M Bauchinger[1] and M Werner[3]**

[1]Institute of Radiobiology, GSF-Forschungszentrum für Umwelt und Gesundheit GmbH, Ingolstäder Landstr. 1, D-85764 Neuherberg, Germany; [2]Institute of Pathology, Technische Universität, Ismaninger Str. 22, D-81675 München, Germany; [3]Institute of Pathology, GSF-Forschungszentrum für Umwelt und Gesundheit GmbH, Ingolstädter Landstr. 1, D-85764 Neuherberg, Germany; [4]Institute of Radiation Biology, Ludwig Maximilians Universität, Schillerstr. 42, D-80336 München, Germany

**Summary** Chromosomal copy number changes were investigated in 16 prostate carcinomas, 12 prostatic intraepithelial neoplasias (PIN; 4 low-grade and 8 high-grade) adjacent to the invasive tumour areas, and 5 regional lymph node metastases. For this purpose, comparative genomic hybridization (CGH) was performed and a copy number karyotype for each histomorphological entity was created. CGH on microdissected cells from non-neoplastic glands was carried out on 3 different cases to demonstrate the reliability of the overall procedure. None of the non-neoplastic tissue samples revealed chromosome copy number changes. In PIN areas, chromosomal imbalances were detected on chromosomes 7, 8q, Xq (gains), and on 4q, 5q, 8p, 13q and 18q (losses). In the primary tumours, recurrent (at least 25% of cases) gains on chromosomes 12q and 15q, and losses on 2q, 4q, 5q, Xq, 13q and 18q became apparent. Losses on 8p and 6q as well as gains on 8q and of chromosome 7 were also detected at lower frequencies than previously reported. The pooled CGH data from the primary carcinomas revealed a novel region of chromosomal loss on 4q which is also frequently affected in other tumour entities like oesophageal adenocarcinomas and is supposed to harbour a new tumour suppressor gene. Gains on chromosome 9q and of chromosome 16 and loss on chromosome 13q were observed as common aberrations in metastases and primary tumours. These CGH results indicate an accumulation of chromosomal imbalances during the PIN–carcinoma–metastasis sequence and an early origin of tumour-specific aberrations in PIN areas.
© 2001 Cancer Research Campaign http://www.bjcancer.com

**Keywords:** prostate carcinoma; prostatic intraepithelial neoplasia; tumour progression; comparative genomic hybridization

Comparative genomic hybridization (CGH) is a powerful tool for genome-wide screening of tumours for copy number changes of DNA sequences (Kallioniemi et al, 1992). Application of CGH and fluorescence in situ hybridization (FISH) to primary tumours of prostatic adenocarcinomas revealed consistent changes on chromosomes 7, 8p, 10, 13q, 16, 17 and 18q (Brothman et al, 1994; Macoska et al, 1994; Matsuyama et al, 1994; Joos et al, 1995; Qian et al, 1995; Visakorpi et al, 1995; Bova and Isaacs, 1996; Cher et al, 1996; Huang et al, 1996; Deubler et al, 1997). However, cytogenetic changes in prostatic intraepithelial neoplasias (PIN) which are considered as premalignant lesions and which are often present besides the invasive tumour are only poorly characterized cytogenetically (Alers et al, 1995; Qian et al, 1995; Zitzelsberger et al, 1998). Methodological improvements of approaches combining microdissection and CGH analysis (Kuukasjärvi et al, 1997) were prerequisites for the analysis of such early chromosomal aberrations in premalignant cells of other tumours like cervical carcinoma (Heselmeyer et al, 1996), breast cancer (Aubele et al, 2000b) and oral malignant lesions (Weber et al, 1998). Their application to premaligant lesions in prostate cancer has recently been described (Zitzelsberger et al, 1998; Kim et al, 1999), but for an improved understanding of mechanisms of tumour development and tumour progression more data on cytogenetic changes in thePIN–adenocarcinoma–metastasis sequence are needed.

The present study reports on the results of CGH analysis in 16 prostatic adenocarcinomas, 12 related PINs and 5 lymph node metastases. These investigations are aimed at a delineation of chromosome copy number changes in the non-neoplastic prostatic gland–PIN–invasive carcinoma–metastasis sequence.

*Received 19 January 2000*
*Revised 1 September 2000*
*Accepted 6 September 2000*

*Correspondence to:* H Zitzelsberger

### MATERIAL AND METHODS

### Tissue samples

Formalin-fixed and paraffin-embedded tissue specimens of 16 adenocarcinomas of the prostate and of 5 related lymph node metastases were analysed. 12 intraepithelial neoplasias (4 low-grade and 8 high-grade PINs) adjacent to the primary tumours and non-neoplastic prostatic glands from 3 cases were additionally investigated. The histological classification and grading were performed on H&E-stained sections (Gleason and Mellinger, 1974; Sobin and Wittekind, 1997). Pathohistological data of cases are summarized in Table 1. Serial 5 µm sections of the tissue blocks were used for microdissection of tissue samples. For FISH analysis, consecutive 10 µm sections were analysed. Non-neoplastic glands, PINs and metastases were laser-microdissected and genomic DNA was amplified by DOP-PCR. DNA of extended areas of primary tumours was isolated from manually microdissected sections which provided sufficient DNA amounts for CGH without prior DOP amplification.

### Laser-assisted microdissection

For microdissection, a laser microscope system (P.A.L.M., Wolfratshausen, Germany) was used consisting of a Zeiss Axiovert microscope (Zeiss, Jena, Germany), a pulsed UV-laser (wavelength 337 nm, maximum frequency: 20 pulses per second, pulse duration: 3 nanoseconds), and a computer-controlled micromanipulator. By means of the focussed UV-laser, unwanted cells or tissue areas, surrounding the cells of interest, were destroyed. Isolated cell compartments of 50 to 100 cells were subsequently collected. The cells were transferred into a sterile PCR reaction tube containing 20 μl laser buffer (100 mM Tris/HCl, pH 7.5, 100 μg ml$^{-1}$ proteinase K). Microdissected probes were then heated for 3 h at 55˚C to allow proteolytic digestion and for 8 minutes at 100˚C to inactivate the proteinase K. Samples were stored at –20˚C until use.

### DOP-PCR

DOP-PCR was performed according to a published procedure (Weber et al, 1998; Zitzelsberger et al, 1998). PCR reaction was carried out in a 50 μl reaction volume (3.5 mM $MgCl_2$, 50 mM KCl, 20 mM Tris/HCl, pH 8.4) containing the microdissected and pretreated cells in 20 μl laser buffer, 0.2 mM primer UW4B (5′-CCGACTCGAGNNNNNNATGTGG-3′), and 4 units Taq polymerase. After 40 PCR cycles (initial step for 10 minutes at 94˚C, 5 cycles with a low annealing temperature at 30˚C, 35 cycles with a high annealing temperature at 62˚C and a final extension step), the size of DNA fragments and DNA yields of each reaction were checked by agarose gel electrophoresis. DNA yields were additionally determined by fluorimetric measurements. To avoid contamination of PCR reactions, they were set up in a laminar flow using special aerosol resistant tips. PCR solutions were additionally checked for possible contaminations in PCR reactions without template DNA using gene-specific primers.

### DNA extraction from paraffin-embedded tissue sections

For primary tumours, CGH was performed from paraffin-embedded tissue sections. The DNA was extracted according to standard procedures.

### DNA labelling

Isolated whole genomic tumour DNA and DOP-PCR amplified samples were labelled with biotin-16-dUTP by standard nick translation. As reference DNA, SpectrumRed direct-labelled normal male total human genomic DNA (Vysis Inc, Downers Grove, IL) was used.

### CGH and image analysis

Metaphase preparations for CGH analyses were obtained from peripheral lymphocytes of a healthy male donor according to standard procedures. CGH analysis was performed according to Kallioniemi et al (1992) and du Manoir et al (1993) with modifcations. 600 ng of biotin-16-dUTP labelled DNA and 600 ng of SpectrumRed direct-labelled normal male DNA were simultaneously hybridized with 25 μg unlabelled Cot-1 DNA (Life Technologies Inc, Grand Island, NY) to denatured lymphocyte metaphases for 3 days. Bound biotin-labelled DNA probes were detected by sequential incubations in Cy2-conjugated streptavidin/biotinylated anti-streptavidin (concentration: 10 μg ml$^{-1}$ and 5 μg ml$^{-1}$ in PNM-buffer consisting of PN-buffer plus 5% non-fat dry milk). Between each incubation step, slides were washed twice in PN-buffer (0.1 M sodium phosphate pH 8.0, 0.1% nonidet P-40). To obtain a fluorescence banding pattern, slides were stained with 4′,6′-diamidino-2-phenylindole (DAPI) at a concentration of 0.1 μg ml$^{-1}$ in antifading solution. CGH images were captured by a black/white video CCD camera using on chip integration. The 3 colours were digitized consecutively with specific single colour filter combinations which were automatically changed on a Zeiss Axioplan2 microscope. For processing of captured images, a CGH analysis software from MetaSystems (MetaSystems, Altlussheim, Germany) was used. For one CGH analysis, 10 to 15 examples of each chromosome were measured after DAPI karyotyping of 5 to 10 metaphases. Average ratio profiles were then calculated after automatically scaling the profiles of individual homologous chromosomes of the same length. Average profiles were interpreted according to published criteria (Kallioniemi et al, 1994; Solinas-Toldo et al, 1996) using statistical confidence limits based on *t*-statistics.

### Control experiments

DOP-PCR amplified DNA obtained from non-neoplastic prostatic glands with morphologically normal appearing prostatic epithelium was hybridized in 3 cases (Table 1) with non-amplified reference DNA (SpectrumRed™) to metaphase preparations. In these experiments no chromsomal changes were detected except for gains on chromosomes 1p34–36 and 19. Such regions of frequent artifactual appearance were excluded from further interpretation of data (see discussion). In addition, 4 cases of prostate carcinoma were comprehensively analysed using both DOP-PCR-amplified and non-amplified DNA. This comparison shows no significant differences for chromosomal changes detected by both methods. For additional control of the DOP-PCR approach microdissected normal epithelium present in tissues from other tumour entities like Barrett's adenocarcinoma (Walch et al, 2000) and ductal breast carcinoma (Aubele et al, 2000b) was investigated and revealed also CGH profiles without alterations.

### Fluorescence in situ hybridization (FISH) analysis

FISH analyses with centromere and locus specific probes for c-myc (8q24), cyclin D1 (11q13), and HER-2/*neu* (17q11.2–q12) were performed to validate the CGH findings. Cases with copy number changes on chromosomes 8q, 11q, and 17q, known from the CGH experiments, were selected to validate these changes. Serial 10 μm sections of the tissue blocks were used for FISH analysis. Areas investigated correspond to those examined by CGH. Commercially available DNA probe kits were used for c-myc (Oncor, Gaithersburg, USA) and for centromere 8 (Oncor, Gaithersburg, USA), cyclin D1/centromere 11 (Vysis, Inc; Downers Grove; USA) and HER-2/*neu*/centromere 17 (Vysis, Inc; Downers Grove; USA). Signals from 150–200 tumour cell nuclei per specimen were counted using a confocal laser scanning microscope (Zeiss LSM 510). Nuclei from normal squamous epithelium or lymphocytes deposited separately on the same slide were used as controls for hybridization efficiency and specificity. The criteria established by Hopman et al (1988) were followed for signal enumeration. Amplification of the respective gene locus was considered for nuclei exhibiting at least twice as many locus-specific signals as centromere signals. More than two locus-specific signals accompanied by the same elevated number of centromere signals were considered to be indicative of polysomy. When the proportion of cells with nuclei

© 2001 Cancer Research Campaign without any signal exceeded 20%, the procedure was regarded as insufficient and therefore repeated. For a detailed description of the FISH method used as well as for the evaluation by confocal laser scanning mircoscopy see Aubele et al (1997).

### RESULTS

A summary of chromosomal imbalances detected in 16 adenocarcinomas, 12 PINs and 5 metastases is shown in Figure 1. 3 cases (Table 1) were also investigated for chromosome copy number changes in non-neoplastic prostatic glands for control; normal profiles were obtained in each of these samples.

The average aberration frequency (± SEM) in the 12 PIN areas was 4.3 ± 1.1. In PIN areas, gains were detected on chromosomes 8q (42%), 7 (25%), 16p (25%), 17 (25%), 19 (33%), 20 (25%), whereas losses were found on 13q (25%). Additionally, gains on chromosomes Xq (17%), 12q (17%), 15q (17%), 22 (17%), 1p, 4p, 11q (one case each), as well as losses on chromosomes 4q (17%), 2p, 3p, 3q, 5q, 6q, 8p, 10q, 12q, 18q and the Y chromosome (one case each) became apparent at lower frequencies. The changes showed a distinct heterogenic distribution, however, all of them were detectable in the corresponding primary tumours as well.

An average of 8.5 ± 0.9 gains and losses was detected in the 16 primary tumours. The following aberrations were identified in at least 25% of tumours: gains on 1p33–36 (38%), 12q24 (25%), 15q23–24 (25%), 16p12–13 (69%), 17 (50%), 19 (75%), 20 (50%), 22 (56%) and losses on 2q32 (25%), 4q28 (25%), 5q21 (31%), Xq (25%), 13q22 (56%), 18q21–23 (25%). Losses on 8p and 6q as well as gains on chromosome 7 and 8q were detected at lower frequencies. However, these aberrations occurred in the corresponding primary tumours as well as in PINs and/or metastases (Fig. 1).

The 5 lymph node metastases showed changes affecting the same chromosomal regions in a similar frequency (mean 7.8 ± 1.2). Common aberrations to primary tumours were gains on chromosomes 9q and of chromosome 16 and loss on chromosome 13q.

CGH data were exemplary validated on selected cases (4778/92, 10673/91, 10844/91) using a FISH approach on consecutive 10 μm sections and subsequent laser scanning microscopy (Table 2). Additionally, LOH analysis for D8S137 locus on chromosome 8p was carried out for validation of 8p loss (Table 2). To verify chromosomal gains on 8q21–24 (4778/92 carcinoma and PIN), 11q13 (10673/91 carcinoma and PIN, 10844/91 carcinoma) and 17 (4778/92 carcinoma), locus-specific probes for c-myc (8q24), cyclin D1 (11q13) and HER-2/neu (17q11.2–q12) were applied together with the respective centromere probes. In case 4778/92 an amplification of the c-myc locus could be detected in carcinoma and PIN areas in addition to polysomy of chromosome 17 in carcinoma areas. Cases 10673/91 (carcinoma and PIN) and 10844/91 carcinoma revealed polysomy of chromosome 11 and amplification of the cyclin D1 locus in case 10844/91. No locus-specific amplifications could be detected for the HER-2/neu locus.

### DISCUSSION

Prostate cancer development and progression is supposed to be driven by the accumulation of cytogenetic and molecular genetic alterations. At the histological level, PINs outside the invasive carcinoma are considered as premalignant lesions of prostate cancer (Bostwick and Brawer, 1987; Bonkhoff and Remberger, 1996). In the PIN–carcinoma–metastasis sequence, PIN areas are poorly characterized for chromosomal alterations because they appear as very small cell compartments which can be only studied utilizing either microdissection and subsequent molecular genetic techniques like CGH (Weber et al, 1998; Zitzelsberger et al, 1998; Kim et al, 1999) or FISH on paraffin sections (Alers et al, 1995; Qian et al, 1996; Jenkins et al, 1997). In this study, we were able to demonstrate cytogenetic changes in 5 lymph node metastases and 12 PIN areas from 6 different cases. Chromosomal imbalances occurring in primary tumours were basically consistent with changes in PINs and metastases, and affected the same chromosomal regions (Fig. 1).

Common changes in the PIN–carcinoma–metastasis sequence became apparent and comprise losses on chromosomes 4q, 5q, 8p, 13q and gains on chromosomes 7, 8q, 12q and 15q. Losses on 2q and gains on 9/9q were only present in carcinoma and metastasis specimens and, thus, may indicate late events during tumorigenesis. Our data set on chromosomal changes in PIN areas provides clues that alterations reported as typical changes in prostate cancer

**Table 1** Prostatic adenocarcinomas, PINs and lymph node metastases analysed

| Case | Gleason Score | pTNM Classification[a] | Age | Tissue lesions analysed |
|---|---|---|---|---|
| 15075/90 | 8 | T3bN0 | 65 | PT |
| 635/91 | 6 | T3bN1 | 64 | PT, Met |
| 5573/91 | 6 | T3b N0 | 63 | PT |
| 5640/91 | 5 | T3a N0 | 67 | PT |
| 9350/91 | 8 | T2b N0 | 52 | PT |
| 14323/91 | 7 | T2b N2 | 67 | Met |
| 15008/91 | 7 | T3b N1 | 62 | N, PIN high grade (3x), PT, Met |
| 862/92 | 9 | T4 N0 | 71 | PIN high grade (2x), PT |
| 1287/92 | 5 | T3a N1 | 73 | PIN low grade, PT, Met |
| 4778/92 | 7 | T3b N2 | 82 | N, PIN low grade, PIN high grade, PT, Met |
| 7757/92 | 4 | T3a N0 | 65 | PT |
| 8039/92 | 9 | T3b N0 | 66 | PT |
| 8385/93 | 3 | T2a N0 | 71 | N, PIN low grade (2x), PT |
| 9971/93 | 8 | T2a N0 | 61 | PIN high grade (2x), PT |
| 10601/93 | 5 | T3a N0 | 67 | PT |
| 7632/94 | 5 | T2a N0 | 55 | PT |
| 14624/94 | 6 | T3a N0 | 70 | PT |

[a]UICC/TNM-Classification (Sobin and Wittekind 1997). N = Non-neoplastic prostatic glands; PIN = Prostatic intraepithelial neoplasia; PT = Primary tumour; Met = Lymph node metastasis.

*© 2001 Cancer Research Campaign*

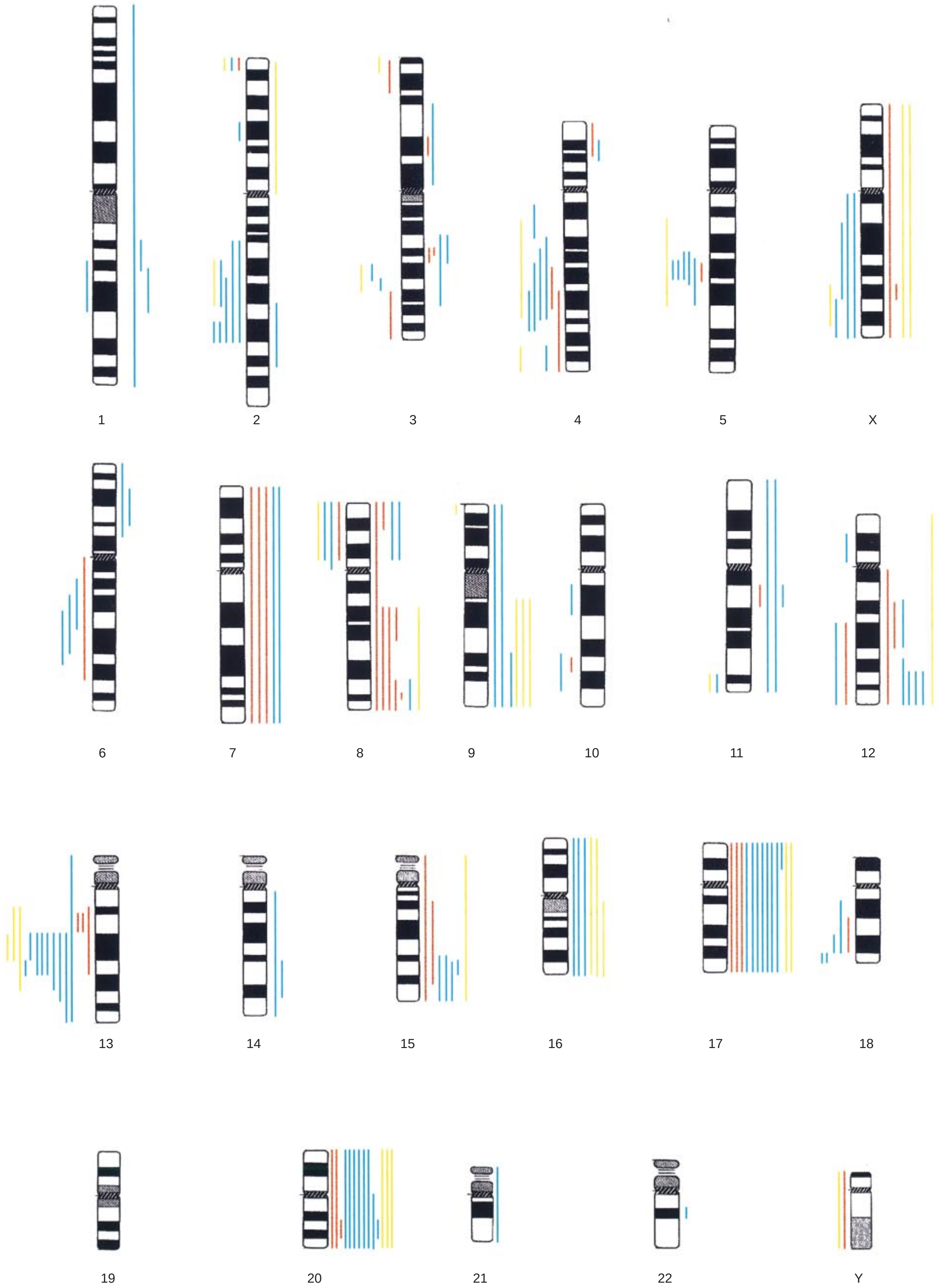

**Figure 1** Chromosomal gains and losses in 12 prostatic intraepithelial neoplasias (orange), 16 prostatic adenocarcinomas (blue) and 5 lymph node metastases (yellow). Gains are indicated on the right side, losses on the left side of ideograms

© 2001 Cancer Research Campaign

**Table 2** Confirmation of CGH results with FISH and LOH analysis

| Case | Representative CGH findings | FISH analysis[1] CEP 8 | c-myc | FISH analysis[1] CEP 17 | HER-2/*neu* | FISH analysis[1] CEP 11 | cyclin D1 | LOH analysis on 8p* |
|---|---|---|---|---|---|---|---|---|
| | | signals per cell[#] (range) | | signals per cell[#] (range) | | signals per cell[#] (range) | | |
| 4778/92 CA | +8q21-24, –8p21-23 | 2.4 (1–3) | 5.0 (2–6) | 2.3 (1–3) | 2.0 (1–3) | nd | nd | AL |
| 4778/92 PIN | +8q21-24, –8p21-23 | 1.9 (1–3) | 4.2 (2–4) | 1.7 (1–3) | 1.9 (1–3) | nd | nd | AL |
| |   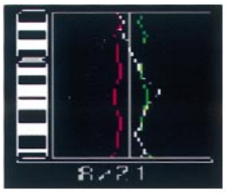 | | | | | | | |
| 10673/91 CA | +11q13, +17 | nd | nd | 3.8 (2–5) | 3.4 (1–4) | 3.1 (1–4) | 2.9 (1–4) | nd |
| 10673/91PIN | – | nd | nd | 2.2 (1–3) | 1.8 (1–3) | 2.2 (1–4) | 1.7 (1–4) | nd |
| |   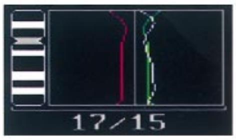 | | | | | | | |
| 10844/9 CA | +11q13-14, +17 | nd | nd | 3.9 (2–5) | 3.6 (1–4) | 2.7 (1–4) | 3.2 (1–4) | AL |
| |   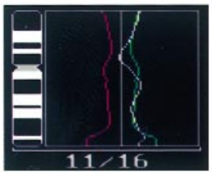 | | | | | | | |

CA = carcinoma. PIN = prostatic intraepithelial neoplasia. CEP = centromeric DNA probe. AL = allelic loss. nd = not determined. * investigation of microsatellite locus D8S137. [1]A gene locus was classified as amplified if there were more than twice locus-specific signals than centromere signals (ratio > 2) per cell nucleus. More than two locus specific signals accompanied by the same number of centromere signals was considered to be indicative of polysomy of the respective chromosome (ratio 1:1). [#]Mean number of signals per cell. CGH profiles: average profiles for chromosomes 8, 11 and 17 are exemplary demonstrated. Below each idiogram, the respective chromosome number (left) as well as the number of homologous chromosomes included in the calculation of the profile (right) are indicated. The red/green ratio is displayed as a white line together with thresholds for loss (red line) and gain (green line). Thresholds are calculated as statistical confidence intervals by the CGH software.

(losses on 8p, 13q, gains on 7, 8q) have a very early origin in PIN. Thus, a subset of PIN areas, irrespective of their differentiation into low- or high-grade PIN, exhibits a number of aberrations similar to invasive carcinoma. These CGH findings on PIN areas confirm for many of the chromosomal alterations earlier studies which investigated corresponding loci either with LOH (Macintosh et al, 1998; Saric et al, 1999) or FISH analysis (Alers et al, 1995; Qian et al, 1996). These data provide evidence for the biological significance of PINs and support the assumption that they represent premalignant lesions of prostate cancer. It might be therefore of prognostic value to survey PIN areas for their chromosomal aberrations.

The most frequent losses (25–56%) in the primary tumours were found on 13q22, 5q21, 2q32, 18q21–23, 4q28 and Xq; most common regions (25–75%) of chromosomal gains were detected on 12q and 15q (Fig. 1). Frequently observed gains on chromosomes 1p, 16p and of whole chromosomes 19 and 22 were not taken into account for the interpretation of data because they are known to represent frequently artifactual results in CGH analysis (Kallioniemi et al, 1994; Lichter et al, 1995; Weber et al, 1998). Frequently occurring gains of whole chromosomes 16, 17 and 20 reflect the aneuploid karyotype of the tumours. The frequent finding of gain on chromosome 12q is in good agreement with a recent publication (Sattler et al, 1999) reporting on frequent copy number gains in human prostate cancer. Losses on 8p and 6q as well as gains on 8q and of chromosome 7, which were considered to be typical aberrations for prostate cancer (for review see Bova and Isaacs, 1996), were also detected in primary tumours investigated in this study, but at a lower frequency. This fact can be partly explained by the smaller number of cases in our study compared to the literature data. The use of laser-ablation of unwanted cells surrounding the carcinoma area prior to CGH analysis might be a second reason for the difference between our and published data because stromal tissue, normal prostatic glands and PIN areas are removed before DNA extraction with this approach. A third difference to most of published prostate cancer cases is the fact that the majority of our cases do not represent advanced stages. Only 5 of 17 cases (29%; Table 1) are metastasizing cancers which might influence the cytogenetic results. With respect to the reported extensive genetic heterogeneity in prostate cancers (Qian et al, 1996; Macintosh et al, 1998) it is not surprising that changes detected in PIN areas coincidentially resemble more to 'typical' published aberrations in prostate cancers than the corresponding primary tumours. Altogether, our findings in primary carcinomas are confirmatory for losses on 13q, 18q, 5q, 2q and gains on 7, 8q.

 *© 2001 Cancer Research Campaign*

In addition to these known hotspots of chromosomal copy number changes in prostatic adenocarcinoma, a novel region of chromosomal loss on 4q could be detected in our subset of cases. Deletions on 4q are frequent events in other tumour entities such as lung tumours (Petersen et al, 1997), renal carcinoma (Jiang et al, 1998), papillary bladder cancer (Simon et al, 1998), and appear to play a crucial role during aggressive progression of hepatocellular carcinoma (Piao et al, 1998). It is postulated that several, yet unidentified, putative tumour suppressor genes may be located on 4q (Hammoud et al, 1996). Although for other known altered chromosomal regions in prostate cancer (8p, 13q, 8q) candidate tumour suppressor genes and oncogenes have already been identified (multiple novel genes on the short arm of chromosome 8, *RB* gene on 13q14, *c-MYC* gene on 8q24), the identification of candidate genes in further chromosomal regions affected is still in its initial stage (Bova and Isaacs, 1996).

In summary, CGH analysis of 16 adenocarcinomas of the prostate revealed a series of known chromosomal imbalances in addition to a novel described loss of DNA sequences on chromosome 4q. Investigation of related PINs and lymph node metastases demonstrated an accumulation of chromosomal imbalances during cancer development and progression and an early origin of tumour-specific aberrations in PIN areas.

### ACKNOWLEDGEMENTS

We are grateful to Mrs S Schulte-Overberg, Mrs A Schreier and Mrs D Angermeier for excellent technical assistance.

### REFERENCES

Alers JC, Krijtenburg PJ, Vissers KJ, Bosman FT, van der Kwast TH and van Dekken H (1995) Interphase cytogenetics of prostatic adenocarcinoma and precursor lesions: analysis of 25 radical prostatectomies and 17 adjacent prostatic intraepithelial neoplasias. *Genes Chrom Cancer* **12**: 241–250

Aubele M, Zitzelsberger H, Szücs S, Werner M, Braselmann H, Hutzler P, Rodenacker K, Lehmann L, Minkus G and Höfler H (1997) Comparative FISH analysis of numerical chromosome 7 abnormalities in 5-micron and 15-micron paraffin-embedded tissue sections from prostatic carcinoma. *Histochem Cell Biol* **107**: 121–126

Aubele MM, Cummings MC, Mattis AE, Zitzelsberger HF, Walch AK, Kremer M, Höfler H and Werner M (2000a) Accumulation of chromosomal imbalances during the development and progression of ductal breast cancer. *Mol Diagn Pathol* **9**: 14–19

Aubele M, Mattis A, Zitzelsberger H, Walch A, Kremer M, Welzl G, Höfler H and Werner M (2000b) Extensive ductal carcinoma in situ with small foci of invasive ductal carcinoma: evidence of genetic resemblance by CGH. *Int J Cancer* **85**: 82–86

Bonkhoff H and Remberger K (1996) Differentiation pathways and histogenetic aspects of normal and abnormal prostatic growth: a stem cell model. *Prostate* **28**: 98–106

Bova GS and Isaacs WB (1996) Review of allelic loss and gain in prostate cancer. *World J Urol* **14**: 338–346

Bostwick DG and Brawer MK (1987) Prostatic intraepithelial neoplasia and early invasion in prostate cancer. *Cancer* **59**: 788–794

Brothman AR, Watson MJ, Zhu XL, Williams BJ and Rohr LR (1994) Evaluation of 20 archival prostate tumor specimens by fluorescence in situ hybridization (FISH). *Cancer Genet Cytogenet* **75**: 40–44

Cher ML, Bova GS, Moore DH, Small EJ, Carroll PR, Pin SS, Epstein JI, Isaacs WB and Jensen RH (1996) Genetic alterations in untreated metastases and androgen-independent prostate cancer detected by comparative genomic hybridization and allelotyping. *Cancer Res* **56**: 3091–3102

Deubler DA, Williams BJ, Zhu XL, Steele MR, Rohr LR, Jensen JC, Stephenson RA, Changus JE, Miller GJ, Becich MJ and Brothman AR (1997) Allelic loss detected on chromosomes 8, 10, and 17 by fluorescence in situ hybridization using single-copy P1 probes on isolated nuclei from paraffin-embedded prostate tumors. *Am J Pathol* **150**: 841–850

du Manoir S, Speicher MR, Joos S, Schröck E, Popp S, Dohner H, Kovacs G, Robert-Nicoud M, Lichter P and Cremer T (1993) Detection of complete and partial chromosome gains and losses by comparative genomic in situ hybridization. *Hum Genet* **90**: 590–610

Gleason DF and Mellinger GT (1974) Prediction of prognosis for prostatic adenocarcinoma by combined histological grading and clinical staging. *J Urol* **111**: 58–64

Hammoud ZT, Kaleem Z, Cooper JD, Sundaresan RS, Patterson GA and Goodfellow PJ (1996) Allelotype analysis of esophageal adenocarcinomas: evidence for the involvement of sequences on the long arm of chromosome 4. *Cancer Res* **56**: 4499–4502

Heselmeyer K, Schröck E, du Manoir S, Blegen H, Shah K, Steinbeck R, Auer G and Ried T (1996) Gain of chromosome 3q defines the transition from severe dysplasia to invasive carcinoma of the uterine cervix. *Proc nat Acad Sci (Wash)* **93**: 479–484

Hopman AH, Ramaekers FC, Raap AK, Beck JL, Devilee P, van der Ploeg M and Vooijs GP (1988) In situ hybridization as a tool to study numerical chromosome aberrations in solid bladder tumors. *Histochemistry* **89**: 307–316

Huang SF, Xiao S, Renshaw AA, Loughlin KR, Hudson TJ and Fletcher JA (1996) Fluorescence in situ hybridization evaluation of chromosome deletion patterns in prostate cancer. *Am J Pathol* **149**: 1565–1573

Jenkins RB, Qian J, Lieber MM and Bostwick DG (1997) Detection of c-myc oncogene amplification and chromosomal anomalies in metastati prostatic carcinoma by fluorescence in situ hybridization. *Cancer Res* **57**: 524–531

Jiang F, Moch H, Richter J, Egenter C, Gasser T, Bubendorf L, Gschwind R, Sauter G and Mihatsch MJ (1998) Comparative genomic hybridization reveals frequent chromosome 13q and 4q losses in renal carcinomas with sarcomatoid transformation. *J Pathol* **185**: 382–388

Joos S, Bergerheim US, Pan Y, Matsuyama H, Bentz M, du Manoir S and Lichter P (1995) Mapping of chromosomal gains and losses in prostate cancer by comparative genomic hybridization. *Genes Chrom Cancer* **14**: 267–276

Kallioniemi A, Kallioniemi OP, Sudar D, Rutovitz D, Gray JW, Waldman F and Pinkel D (1992) Comparative genomic hybridization for molecular cytogenetic analysis of solid tumours. *Science* **258**: 818–821

Kallioniemi OP, Kallioniemi A, Piper J, Isola J, Waldman FM, Gray JW and Pinkel D (1994) Optimizing comparative genomic hybridization for analysis of DNA sequence copy number changes in solid tumours. *Genes Chrom Cancer* **10**: 231–243

Kim SH, Godfrey T and Jensen RH (1999) Whole genome amplification and molecular genetic analysis of DNA from paraffin-embedded prostate adenocarcinoma tumor tissue. *J Urol* **162**: 1512–1518

Kuukasjärvi T, Tanner M, Pennanen S, Karhu R, Visakorpi T and Isola J (1997) Optimizing DOP-PCR for universal amplification of small DNA samples in comparative genomic hybridization. *Genes Chrom Cancer* **18**: 94–101

Lichter P, Bentz M, du Manoir S and Joos S (1995) Comparative genomic hybridization. In: Verma RS and Babu A (eds) *Human Chromosomes*, New York: McGraw Hill, pp 191–210

Macintosh CA, Stower M, Reiid N, Maitland NJ (1998) Precise microdissection of human prostate cancers reveals genotypic heterogeneity. *Cancer Res* **58**: 23–28

Macoska JA, Trybus TM, Sakr WA, Wolf MC, Benson PD, Powell IJ and Pontes JE (1994) Fluorescence in situ hybridization analysis of 8p allelic loss and chromosome 8 instability in human prostate cancer. *Cancer Res* **54**: 3824–3830

Matsuyama H, Pan Y, Skoog L, Tribukait B, Naito K, Ekman P, Lichter P and Bergerheim US (1994) Deletion mapping of chromosome 8p in prostate cancer by fluorescence in situ hybridization. *Oncogene* **9**: 3071–3076

Nupponen NN, Kakkola L, Koivisto P and Visakorpi T (1998) Genetic alterations in hormone-refractory recurrent prostate carcinomas. *Am J Pathol* **153**: 141–148

Petersen I, Langreck H, Wolf G, Schwendel A, Psille R, Vogt P, Reichel MB, Ried T and Dietel M (1997) Small-cell lung cancer is characterized by a high incidence of deletions on chromosomes 3p, 4q, 5q, 10q, 13q, and 17p. *Br J Cancer* **75**: 79–86

Piao Z, Park C, Park JH and Kim H (1998) Deletion mapping of chromosome 4q in hepatocellular carcinoma. *Int J Cancer* **79**: 356–360

Qian J, Bostwick DG, Takahashi S, Borell TJ, Herath JF, Lieber MM and Jenkins RB (1995) Chromosomal anomalies in prostatic intraepithelial neoplasia and carcinoma detected by fluorescence in situ hybridization. *Cancer Res* **55**: 5408–5414

Qian J, Bostwick DG, Takahashi S, Borell TJ, Brown JA, Lieber MM and Jenkins

© 2001 Cancer Research Campaign 

RB (1996) Comparison of fluorescence in situ hybridization analysis of isolated nuclei and routine histological sections from paraffin-embedded prostatic adenocarcinoma specimens. *Am J Pathol* **149**: 1193–1199
Saric T, Brkanac Z, Troyer DA, Padalecki SS, Sarosdy M, Williams K, Abadesco L, Leach RJ and O'Connell P (1999) Genetic pattern of prostate cancer progression. *Int J Cancer* **81**: 219–224
Sattler HP, Rohde V, Bonkhoff H, Zwergel T and Wullich B (1999) Comparative genomic hybridization reveals DNA copy number gains to frequently occur in human prostate cancer. *Prostate* **39**: 79–86
Simon R, Burger H, Brinkschmidt C, Bocker W, Hertle L and Terpe HJ (1998) Chromosomal aberrations associated with invasion in papillary superficial bladder cancer. *J Pathol* **185**: 345–351
Sobin LH and Wittekind CH (1997) International Union against Cancer (UICC) TNM-Classification of malignant tumor 1997. New York: Wiley-Liss
Solinas-Toldo S, Wallrapp C, Muller-Pillasch F, Bentz M, Gress T and Lichter P (1996) Mapping of chromosomal imbalances in pancreatic carcinoma by comparative genomic hybridization. *Cancer Res* **56**: 3803–3807
Visakorpi T, Kallioniemi AH, Syvänen AC, Hyytinen ER, Karhu R, Tammela T, Isola JJ and Kallioniemi OP (1995) Genetic changes in primary and recurrent prostate cancer by comparative genomic hybridization. *Cancer Res* **55**: 342–347
Walch AK, Zitzelsberger HF, Bruch J, Keller G, Angermeier D, Aubele MM, Mueller J, Stein H, Braselmann H, Siewert JR, Höfler H and Werner M (2000) Chromosomal imbalances in Barrett's adenocarcinoma and the metaplasia-dysplasia-carcinoma sequence. *Am J Pathol* **156**: 555–566
Weber RG, Scheer M, Born IA, Joos S, Cobbers JM, Hofele C, Reifenberger G, Zoller JE and Lichter P (1998) Recurrent chromosomal imbalances detected in biopsy materialfrom oral premalignant and malignant lesions by combined tissue microdissection, universal DNA amplification, and comparative genomic hybridization. *Am J Pathol* **153**: 292–303
Zitzelsberger H, Kulka U, Lehmann L, Walch A, Smida J, Aubele M, Lörch T, Höfler H, Bauchinger M and Werner M (1998) Genetic heterogeneity in a prostatic carcinoma and associated prostatic intraepithelial neoplasia as demonstrated by combined use of laser-microdissection, degenerate oligonucleotide primed PCR and comparative genomic hybridization. *Virchows Arch* **433**: 297–304

 *© 2001 Cancer Research Campaign*
